# Psychobiotic Effects of Postbiotics in Depression, Psychosis and Mania

**DOI:** 10.34172/hpp.44340

**Published:** 2026-06-06

**Authors:** Aziz Homayouni-Rad, Jalil Houshyar, Hossein Alikhah, Saba Kamalledin Moghadam, Zohreh Osouli, Ali Asgharzadeh, Vahideh Sarabi-Aghdam, Alireza Asghari

**Affiliations:** ^1^Student Research Committee, Tabriz University of Medical Sciences, Tabriz, Iran; ^2^Department of Food Science and Technology, Faculty of Nutrition and Food Sciences, Tabriz University of Medical Sciences, Tabriz, Iran; ^3^Endocrine Research Center, Tabriz University of Medical Sciences, Tabriz, Iran; ^4^Emergency Medicine Specialist, Assistant Professor, Maragheh University of Medical sciences, Maragheh, Iran; ^5^Department of Food Science and Technology, National Nutrition and Food Technology Research Institute, Shahid Beheshti University of Medical Sciences, Tehran, Iran; ^6^Mental Health Unit, Health Vice Chancellor, Maragheh University of Medical Sciences, Maragheh, Iran; ^7^Department of Clinical Psychology, School of Medicine, Tabriz University of Medical Sciences, Tabriz, Iran; ^8^Canadian College of Integrative Medicine, Canada

**Keywords:** Anxiety disorders, Bipolar disorder, Depressive disorder, Gastrointestinal microbiome, Mood disorders, Probiotics

## Abstract

**Background:**

The gut microbiota significantly influences mental health through the gut-brain axis, modulating mood, cognition, and emotional regulation. While probiotics and prebiotics have been widely studied for their psychobiotic effects, postbiotics—metabolic byproducts of probiotics—represent an underexplored area with potential therapeutic applications. Understanding the role of postbiotics in mental health disorders, such as depression, psychosis, and mania, could lead to novel treatment strategies. This review examines the psychobiotic potential of postbiotics and their mechanisms of action.

**Methods:**

A systematic literature review was conducted to evaluate the effects of postbiotics on mental health conditions. We searched PubMed, Medline, EMBASE, and the Cochrane Library for English-language articles published between January 1, 2015, and January 1, 2025, using keywords such as "postbiotic," "paraprobiotic," "depression," "anxiety," and "psychosis." A manual search supplemented the electronic search to ensure comprehensive coverage. Studies focusing on postbiotic effects on mood regulation, neuroinflammation, and neurotransmitter modulation were included.

**Results:**

Postbiotics demonstrate promising psychobiotic effects in depression, psychosis, and mania. They modulate neurotransmitter levels, including serotonin and gamma-aminobutyric acid (GABA), and reduce neuroinflammation, contributing to improved mood and cognitive function. Additionally, postbiotics influence the hypothalamic-pituitary-adrenal (HPA) axis, enhancing stress response and emotional regulation. These findings suggest that postbiotics may serve as effective therapeutic agents for mental health disorders.

**Conclusion:**

Postbiotics offer significant potential as novel interventions for mental health conditions, with mechanisms involving neurotransmitter modulation and neuroinflammation reduction. Further empirical research is needed to elucidate their clinical applications and optimize therapeutic protocols. This review highlights the importance of postbiotics in advancing innovative strategies to improve mental health outcomes and well-being.

## Introduction

 Mental health disorders represent a major global public health challenge, affecting a significant portion of the population. The World Health Organization reports that over 450 million individuals worldwide suffer from conditions such as depression, anxiety, and psychosis.^1^ These disorders lead to reduced quality of life, impacting individuals, families, and communities. The increasing prevalence of stress-related conditions highlights the urgent need for effective interventions. Addressing mental health is essential for improving global well-being and reducing societal burdens. This pressing issue underscores the importance of innovative therapeutic approaches. Recent research has identified the gut-brain axis as a critical pathway influencing mental health. The gut microbiota plays a key role in modulating brain function, including mood and cognition.^2^ Dysbiosis, or microbial imbalance, can contribute to neurological and behavioral dysfunctions, exacerbating conditions like depression and anxiety.^2^ This bidirectional communication offers a novel perspective on mental health treatment. Exploring microbial interventions could lead to new strategies for managing these disorders. The gut-brain axis provides a foundation for investigating microbiota-based therapies.

 Probiotics, defined as beneficial live microorganisms, have shown potential in supporting mental health when consumed in adequate amounts,^3^ as presented in Table 1. However, emerging evidence suggests that postbiotics, the metabolic byproducts of probiotics, may offer even greater benefits for mental health.^4-9^ These compounds, including short-chain fatty acids, enzymes, and biogenic amines, reduce inflammation and enhance intestinal health.^10-14^ Probiotic-rich foods such as yogurt, kefir, sauerkraut, and kimchi, as well as fortified products, serve as sources of postbiotics.^15-19^ Additionally, postbiotics can be taken indirectly through the consumption of probiotic-rich foods or supplements. In addition, some foods and beverages are fortified with postbiotics.^20-22^ The therapeutic potential of postbiotics makes them a promising focus for mental health research. Their ability to modulate neurotransmitter activity warrants further exploration.

**Table 1:** The Effect of probiotics on mental illness

**Disorders**	**Probiotic**	**Result**	**Reference**
Alzheimer’s Disease	*S.thermophilus* DSM 32245 *B.lactis* DSM 32246, *B. lactis* DSM 32247 *L. acidophilus* DSM 32241 *L.helveticus* DSM 32242, *L. paracasei* DSM 32243 *L. plantarum* DSM 32244 *L.brevis* DSM 27961	Increasing glucose transporters and insulin-like growth factor receptor β modulation of the pAktand pAMPK pathways that lead to decreased hyperphosphorylation of tau protein	^23^
Parkinson’s Disease	*Lactobacillus casei Shirota*(in fermented milk)	Gut microbiota changes associated with intestinal inflammation may contribute to the initiation of α-synmisfolding bacterial proteins may elicit cross-seeded misfolding, inflammation and oxidativestress, and cellular toxicity in neurodegeneration	^24^
Multiple Sclerosis	*Lactobacillus acidophilus, Lactobacillus casei, Bifidobacteriumbifidum*and* Lactobacillus fermentum*	the induction of experimental autoimmune encephalomyelitis (EAE), by myelin oligodendrocyteglycoprotein peptide,	^25-27^
Autism	*Lactobacilus acidophilus* *comprising Lactobacilli, Bifidobacilli, and* * Streptococcus species*	Less diverse fecal microbiome by pyrosequencing of 16S rDNA in children suffering autism, decreasing Firmicutes, Bacteriodetes, and Actinobacteria	^28-30^
Schizophrenia	increased abundance of *Lactobacilli and Actinobacteria*	the disorder is associated with changes in Gammaproteobacteria at class level, Enterobacteriales at order level, and Bacteroidesfragilis at species level a panel consisting of Aerococcaceae, Bifidobacteriaceae, Brucellaceae, Pasteurellaceae, and Rikenellaceae is sufficient to distinguish patients from controls decreased abundance of γ-Proteobacteria and Bacteroidetes	^31,32^
Alcohol Dependence	heat-killed *L. brevis*SBC8803 Yakult 400	maintaining tight junction protein expression, improving intestinal villus/crypt histology, changing mucus thickness, normalizing intestinal cytokines levels and balancing intestinal immunity increased expression of cytoprotective Hsp25 mRNA in the small intestine and prevented ethanolinduced overexpression of TNF Clostridium coccoides ↑ Bacteroidesfragilis ↑ Enterobacteriaceae ↓ E.coli ↓	^33,34^
ADHD	*L. rhamnosus* GG *Lactobacillus rhamnosus*GG ATCC53103 (LGG)	↓risk for ADHD children and adolescents with ADHD who received LGG supplementation reported better health-related QoL compared to their peers who received the placebo	^35,36^

 This review synthesizes current evidence on the psychobiotic effects of postbiotics in depression, psychosis, and mania. A systematic search was conducted across PubMed, Medline, EMBASE, and the Cochrane Library, using keywords like “postbiotic,” “paraprobiotic,” “depression,” and “anxiety.” The search targeted English-language articles published between January 1, 2015, and January 1, 2025, supplemented by a manual search for comprehensive coverage. Key findings are summarized in Table 2, outlining postbiotic effects on selected mental health conditions. Further research is needed to establish postbiotics as viable treatments. This study aims to contribute to innovative mental health solutions by highlighting current evidence and research gaps.

**Table 2:** The Effect of postbiotics on mental illnesses.

**Bacteria**	**Form of probiotic**	**Research model**	**Dosage**	**Results**	**References**
*Enterococcusfaecalisstrain EC-12 (EC-12)*	Heat killed	Mice	diet enriched with 0.125% concentration of postbiotic	↓ anxiety-like and depression-like behavior	^37^
*Lactobacillus* *fermentum and Lactobacillus delbrueckii*	Heat-killed	Mouse model.	(3 _ 109 cell bodies/gram of chow) for three weeks	↓ locomotor activity in the open-field test and reducing the baseline corticosterone Levels	^38^
*Lactobacillus paracasei,*	Heat-killed	Mice	1 _ 1010 12 weeks	↓Stress – no adverse effect of postbiotic observed	^39^
*Lb. gasseri CP2305*	Postbiotic	Human	(1 × 1010 cfu/day) for 24 days	↓STAI scores after intervention	^40^
*Lacticaseibacillus paracasei NK112*	Heat killed	Mice	-	↓ K1-induced anxious, depressive, and memory-impaired behaviors - elevated Y-maze tasks, IL-1β, IL-6, and tumor necrosis factor (TNF)-α expression	^41^
*Lb. gasseri CP2305*	Paraprobiotic	Human	1 × 1010 cfu/day daily for 12 weeks	Suppressed STAI escalation	^42^
*Lactobacillus helveticus strain MCC1848*	heat-killed	Mice	1 × 1010 cfu/day 24 weeks	↓Anxiety- or depressive-like behaviors in sCSDS mice.	^43^
*Lactobacillus paracasei PS23*	Heat killed	Mice	-	↓ Protein levels of brain-derived neurotropic factor, mineralocorticoid, and, glucocorticoid receptors in the hippocampus dopamine levels in hippocampus and prefrontal cortex.	^44^
*Lactobacillus helveticus*	Heat killed	Human	5*10^9^ cfu/day for 4 weeks	↑the shortened version of the POMS 2 ‘friendliness’ and the VAS ‘relaxed -Had no significant effects on negative mood state items (e.g. anger, nervousness, and confusion)	^45^

## Role of postbiotics in depression

**Figure 1:**
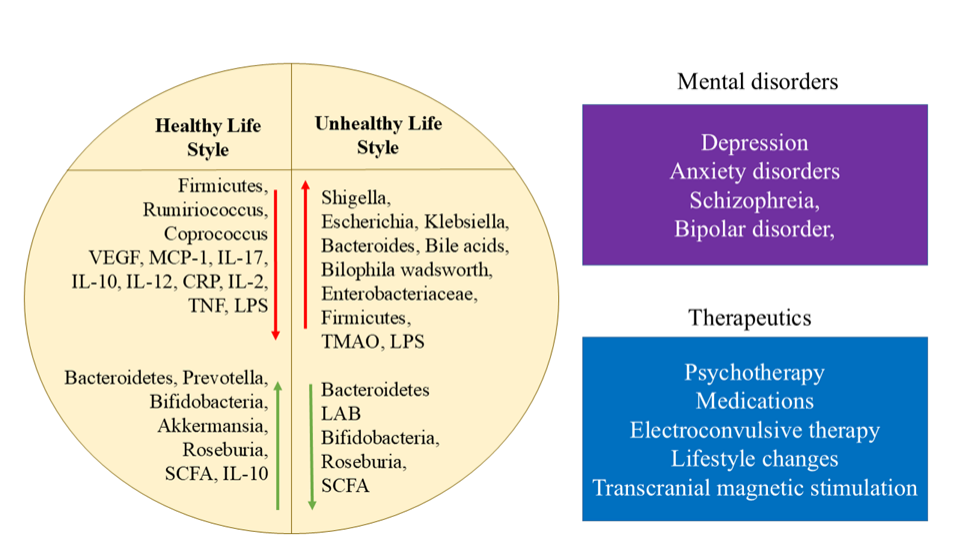
Effects of a healthy lifestyle (regular exercise, a balanced diet, adequate sleep, and stress management) versus unhealthy lifestyles (poor nutrition, lack of physical activity, inadequate sleep, and high stress) on mental health

 Depressive and anxiety disorders rank among the most prevalent mental health conditions affecting individuals. Variants in the oxytocin receptor and serotonin transporter genes have all been associated with anxiety disorders.^46,47^ In depression, brain neurotransmitters such as dopamine, serotonin, and norepinephrine are reduced.^48^ Depression, characterized by a persistently low mood and loss of interest, is often accompanied by feelings of guilt, despair, loss of appetite, and insomnia. It is a prevalent type of mental disorder.^49^ Anxiety is an emotional condition marked by feelings of stress and fear, accompanied by physical symptoms like nervousness, trembling, and issues related to digestion, breathing, and circulation, often occurring without any clear external triggers.^50^ According to the World Health Organization,^51^ 280 million people have depression worldwide. Depression was approximately 50% more common among women than among men in 2023.^51^ Depression, anxiety, and stress are gradual processes of emotional deterioration. Stress can result not only from physiological or emotional challenges but also from temporary reactions in fast-paced situations. Multiple factors influence mood status in human beings. Several studies have confirmed a meaningful association between depression and factors such as neurotransmitter imbalances, a positive family history, stressful life events, and hormonal changes.^52^ Treatments for depression and anxiety basically include medication, psychotherapy, and lifestyle changes (as shown in Figure 1). There is a complicated relationship between anxiety and human depression.^53^ Anxiety, acute and chronic stress, and depression cause changes in the gut microbial profile.^54^ Measuring of brain-derived neurotrophic factor (BDNF) levels,^55^ and adrenocorticotropic hormone and cortisol levels,^56^ indicated a connection between depression and anxiety. Prolonged exposure to stress results in elevated cortisol levels, which can diminish the availability of other neurotransmitters in the brain, such as dopamine and serotonin, both of which are linked to depression.^57^ BDNF serves as a crucial element connecting stress to depression.^58^ Several clinical and animal studies have demonstrated that stress diminishes the expression and activity of brain-derived neurotrophic factor (BDNF) in the hippocampus. This change mirrors the alterations seen in individuals with monoamine deficiency.^59-61^ Additionally, the management of stress is linked with the gut microbiota, which is responsible in the stress response.^62^

 Psychological stresses activate the neuroendocrine, nervous, and immune systems and alter mood and the gut microbiota.^63^ The gut microbiota (GM) plays a crucial role in both the development and functioning of the gut-brain axis, influencing it in significant ways.^64^ Disruption of the gut microbiota may contribute to the development of mental health and neurological disorders.^65^ Another study investigated the gut microbiota of individuals diagnosed with depression, comparing it to that of a control group through fecal microbiota analysis. The findings revealed significant differences between the two groups. Notably, *Lachnospiraceae* was found to be less prevalent, while *Bacteroidaceae* was more abundant in the feces of those with depression.

 Additionally, a specific clade within Alistipes was linked to depressive symptoms. Diets high in carbohydrates and simple sugars were shown to promote the growth of this clade in the gut.^66^ Valeric acid, which is a metabolic byproduct of this bacterial genus, is recognized as a neurochemical compound that interacts with GABA-α receptors.^67^ The impact of *L. rhamnosus* on neurotransmission was studied in mice, which revealed that this strain selectively regulates the expression of gamma-aminobutyric acid (GABA) within the brain. and ultimately reduces depression-related behavior. A comparison of the results in vagotomized mice revealed the absence of these effects. Therefore, the vagus nerve must play an imperative role in mediating the influences of the gut on the brain.^68^ These findings are consistent with those of another study that examined the use of direct vagus nerve stimulation (VNS) as a treatment for depression that does not respond to traditional antidepressants.^69^ Furthermore, the clinical trial conducted by Wallace and Milev^70^ was to evaluate the effects of probiotics on anxiety and depression symptoms of Major Depressive Disorder (MDD). The result of consummation of probiotic supplements containing *Lactobacillus helveticus* R0052 and *Bifidobacterium longum* R0175 at a dose of 3 × 10^9^ CFU once per day for 8 weeks revealed substantial improvements in affective clinical symptoms were observed. In another study, anxiety-like behaviors were diminished in *B. dentium-*monocolonized mice compared with control mice, likely because of the stimulation of serotonin synthesis and the regulation of serotonin transporters (SERTs) in the gastrointestinal tract and the receptors present both in the gut and the brain.^71^ Probiotics enhance tryptophan levels through the serotonin pathway by decreasing the activity of enzymes that convert tryptophan into kynurenine.^72^ Therefore, reducing the kynurenine/tryptophan ratio in the probiotic group may be a mechanism for the detected effects on depression.^73^ In 2017, Colica et al.^74^ performed a clinical trial on humans using a psychobiotic suspension containing *Lactobacillus bulgaricus (CNCM strain numbers I-1632 and I-1519), Lactobacillus subsp. lactis *and* Streptococcus thermophilus *and reported a considerable lessening in anxiety parameters (HAM-A) in people with depressive symptoms. Similarly, 30 days of intake of a probiotic mixture containing *L. helveticus*and *B. longum* had valuable consequences on anxiety and depressive measures and resulted in lowered concentrations of the stress hormone cortisol in individuals without health issues.^75^ Heat-killed *Lactobacillus plantarum* effectively stopped sexually transmitted (ST) infection in mice, demonstrated by reduced weight loss, bacterial translocation, and tissue damage. The LP probiotics significantly mitigated brain injury and neuroinflammation, as indicated by lower levels of interleukin (IL)-1β and IL-6, alongside higher levels of IL-4 and IL-10. Behavioral assessments showed that metabolites from the protected mice countered ST-induced anxiety, depressive-like symptoms, and cognitive decline.^76^

## Role of postbiotics in psychosis

 Psychosis is a mental health condition categorized by a loss of contact with reality. This can manifest as delusions, hallucinations, disorganized thinking and speech, and difficulty in distinguishing between what is real and what is not. Schizophrenia spectrum disorder that includes schizoaffective disorder (depressed type), schizotypal personality disorder, schizophreniform, and atypical psychosis.^77^ Importantly, psychosis can be a symptom of other mental health conditions or medical illnesses, and it is vital to pursue professional help for an accurate diagnosis and suitable treatment.^78^

 Therapies that modulate oxytocinergic signaling have regulating effects on anxiety, depression, and schizophrenia and may offer new policies for the treatment of mental health disease and social behavior.^76,79^

 Positive symptoms are those characterized by the existence of an abnormal phenomenon and include delusions, hallucinations, thought disorder, bizarre behavior, and catatonia.^77^ These effects are thought to result from an overabundance of dopamine in the mesolimbic pathway.^80^ Glutamate is recognized as an excitatory neurotransmitter, and research has indicated a reduction in the functionality of the N-methyl-D-aspartate (NMDA) glutamate receptor as well as gamma-amino-butyric acid (GABA), a substantial inhibitory neurotransmitter.^81^ Studies indicate that dysfunction in patients suffering from schizophrenia is associated with an imbalance in acetylcholine.^82^ The management of patients with psychotic symptoms varies markedly, depending on the underlying etiological and pathophysiological factors contributing to the psychosis, which may include neuromodulatory pathways. Antipsychotic medications are typically the standard treatment for managing psychotic episodes and disorders. The selection of medication, along with its dosage and method of administration, is primarily determined by the specific circumstances of each case. Probiotic supplementation has constant outcomes on the gut microbiota as well as anti-inflammatory and immune-modulatory properties. Disruptions in gut microbiota are associated with prominent serum levels of pro-inflammatory cytokine,^83^ and there is a relationship between the levels of inflammatory markers and the intensity of psychotic symptoms.^84^ Moreover, taking probiotic supplements could be advantageous for addressing undesirable symptoms in patients with schizophrenia.^85^ It was previously reported that probiotic supplementation markedly increases the levels of brain-derived neurotrophic factor (BDNF).^86^ BDNF has an advanced role in neuroplasticity and neuronal survival,^87^ and alterations in BDNF levels are linked to the cognitive impairments often observed in individuals with chronic schizophrenia.^88^ Furthermore, neurotransmitters that cross the blood‒brain barrier, such as serotonin and γ-aminobutyric acid (GABA), can affect brain function.^85^ Nagamine, Sato et al. (2012) reported that the microbiota of patients with chronic schizophrenia before probiotic usage was characterized by high percentages of Bacteroides, Clostridium, Enterococcus, and Ruminococcus and by a percentage of Bifidobacterium close to zero. Alterations in fecal microbiota and a reduction in negative symptoms were observed following probiotic treatment, highlighting the potential clinical significance of immunomodulation in individuals with schizophrenia.^89^ Equally, Jamilian and Ghaderi (2021) reported that probiotics comprising *Lactobacillus acidophilus*, *Bifidobacterium bifidum*, *Bifidobacterium lactis* and *Bifidobacterium *along with selenium supplementation over a three-month period, positively impacted the general positive and negative syndrome scale (PANSS) scores and improved certain metabolic profiles in patients with chronic schizophrenia.^90^ According to the registered records of ClinicalTrials.gov, several policies have aimed at modifying the neurochemical effects of dopamine, serotonin and oxytocin for the treatment of cognitive impairment in patients with schizophrenia/psychosis.^91,92^

## Role of postbiotics in mania

 Bipolar disorders, also known as manic disorders or manic episodes, refer to a set of mental illnesses characterized by periods of intense and elevated mood, energy, and activity. These episodes are often accompanied by symptoms such as racing thoughts, impulsivity, increased goal-directed activity, increased self-esteem and decreased need for sleep.^93^ Treatments for manic disorders usually involve a mixture of medication, therapy and support services to cope with symptoms and develop quality of life.^94,95^ Manic episodes can be accompanied by intense feelings of happiness and euphoria. Importantly, these feelings are often extreme and can lead to impulsive and risky behavior. It is therefore important to distinguish between genuine happiness and an elevated mood during a manic episode.^96,97^

 Recent studies have indicated that mania may be caused by a combination of environmental and genetic factors, imbalances in brain chemistry, and medical disorders, including central nervous system (CNS) syphilis, delirium, encephalitis, brain tumors, and influenza.^98,99^ Depression is frequently associated with systems that respond to stress,^100^ whereas mania is related to energy and mitochondrial dysfunction.^101^ Studies in a mouse model revealed that nitrated product intervention changed *Lachnospiraceae* and *Erysipelotrichia,* leading to the induction of manic-like behaviors. Therefore, there is a connection between dietary habits, changes in behavior, and the composition of gut microbiota in relation to mania.^102^

 Additionally, the capacity of the gut microbiota to influence ENS function has also been shown to be affected by the effects of probiotics on myenteric neuronal function. Administering *Lactobacillus reuteri* to rats for a period of nine days heightened the excitability of enteric sensory neurons (AH) and reduced the duration of slow AH.^103^ The application of a fermented medium produced by Bifidobacterium longum to an in situ preparation of mouse myenteric plexus neurons resulted in a decrease in the impulsiveness of ENS neurons,^104^ recommending that there might be some specificity in the way that various bacteria affect ENS functions. The administration of *Lactobacillus rhamnosus* to healthy BALB/c mice led to a reduction in mania-like behaviors and induced long-term alterations in gamma-aminobutyric acid receptor expression within the central nervous system (CNS).

 In Swiss Webster (CFW) mice, intraluminal delivery of *L. rhamnosus* resulted in a better firing rate of the mesenteric nerve bundle, suggesting that this probiotic strain activates specific neural pathways. This response was inhibited by subdiaphragmatic vagotomy, highlighting the involvement of the vagus nerve in the effects observed from the probiotic.^105^ An improved mood was shown in healthy volunteers following 3 weeks of consumption of an *L. casei*-containing milk drink.^106^ Additionally, female mice fed *L. reuteri* exhibit commonplace grooming activity than control mice do. Oxytocin, a neurohypophyseal hormone, regulates this aspect of maternal behavior. OXytocin plays an essential role in the effects of the central nervous system on behavior.^107^ Interestingly, it affects the body’s energy balance and the immune system.^108^ The postbiotics of *L. plantarum* provided similar benefits to probiotic bacteria in heat-stressed broilers.^109^ The addition of the cell-free supernatant of *L*. *plantarum* to bird feed increased the percentage of eggs produced by birds under heat stress.^110^ In another study, the use of sodium butyrate (NaB) as a postbiotic improved animal sociability and memory in passive avoidance (PA). Furthermore, coadministration of α-lactalbumin,^46^ at each dose used, with NaB (100 mg/kg) was impressive in generating antidepressant-like activity, declining the immobility time (IT) in treated mouse behavioral models of idiopathic autism (BTBR), which exhibit a 100% absence of the corpus callosum and a strictly reduced hippocampal commissure in comparison with those of the control group.^111^ Changes in serotonin receptor (5-HT1A) expression in the hippocampus and SERT mRNA levels in the gut were not observed in animals that were fed heat-killed bacteria. The clear reduction in 5-HT-mediated neurotransmission after the inactivation of probiotics indicates that this system may be solely affected by the metabolites produced by the probiotic bacteria, known as postbiotics. Contrariwise, the GABA system may be modulated by probiotic cell wall components (as another postbiotic form) that physically interact with the host gut mucosa.^112^ The effects of antidepressants and antipsychotic drugs and the effects of monoamines (e.g., serotonin and dopamine) on mania and bipolar disorder have been documented through biological studies.^113,114^

## How postbiotics manage mood

 The metabolites synthesized by the host microbiota, for example, short-chain fatty are linked to the release of hormones that promote positive emotions and a sense of well-being. They can enhance social interactions and help alleviate symptoms of anxiety, stress, and depression; thus, probiotics and their postbiotics could regulate these hormones and improve life-menial qualities, which refer to the small, everyday qualities and tasks that make up a person’s life.^115^ The role of some postbiotics in mental health is described. Interference with the gut microbiota involves variations in the levels of various neurotransmitters and neuromodulators, primarily serotonin, γ-aminobutyric acid (GABA), and dopamine. These changes are linked to the interactions between gut microbiota and brain function.^116^ The hypothalamic‒pituitary‒adrenal (HPA) axis is another fascinating regulatory system.^117^ (Table 3)

**Table 3:** the Effect of postbiotics/probiotics on happiness hormones

**Bacteria**	**Dosage**	**Research model**	**Result**	**Reference**
*Bifidobacterium infantisBi-26, Lactobacillus rhamnosusHN001, Bifidobacterium lactisBL-04, and Lactobacillus paracaseiLPC-37*	10^10^ CFU/pack For 108 day	Human	↑Serotoninand dopamine ↓The severity of autism and gastrointestinal symptoms.	^118^
*Bacillus clausii and Lactobacillus fermentum NMCC-14)*	10^10^ CFU/day for 14 day	Mouse	↓Blood cortisol and adrenocorticotropic hormone (ACTH) levels ↑The dopamine, serotonin, and norepinephrine levels ↑ expression of mRNA of D1 and D2 (except HC, LF-S, day 14) receptors and synaptophysin	^119^
*Lactobacillus plantarum DR7*	for 12 weeks	Human	↑gene expression of dopamine beta hydrolase (DBH)- tryptophan hydroxylase-II (TPH2, serotonin pathway)	^120^
*B longum DSM 24736, B infantis DSM 24737, B breve DSM 24732, L acidophilus DSM 24735, L paracasei DSM 24733, L bulgaricus DSM 24734, L plantarum DSM 24730, and Streptococcus salivarius subspecies thermophilus DSM 24731*	15/100 mg kg^–1^ for 40 days	Rat	↑the expression of many genes related to neuroplasticity and serotonin receptor (5HT)	^121^
*L. reuter*	3.5 × 10^5^ organisms/mouse/day in drinking water for 7 days	Mouse	Up regulation of oxytocin in female mice ↑ offspring survival for mouse mothers fed LR	^122^
*Lactobacillus plantarum PS128 probiotic*	daily intake of (6 × 10^10^ CFUs) for 28 weeks	Human	There were no significant changes of the four groups on the OXT level	^123^
*Lactobacillus reuteri*	3.5 × 105 organisms/mouse/day for two weeks	Mouse	Enhancement of wound-healing through upregulation of the neuropeptide hormone oxytocin	^124^

###  Mood Management by Short-Chain Fatty Acids

 Cyclopropane fatty acids (CFAs), including acetate, butyrate, and propionate, are produced through the fermentation of indigestible carbohydrates (prebiotics) by anaerobic bacteria in the gut. These short-chain fatty acids (SCFAs) serve as a vital energy source for both the intestinal microbiota and intestinal epithelial cells (IECs), influencing various nonimmune and immune functions within the intestinal environment.^125-127^ In addition, SCFAs maintain the integrity of the intestinal mucosal barrier. Mucin gene expression in goblet cells is upregulated in response to SCFAs.^128^ Additionally, *B. longum *colonization safeguarded mice from fatal outcomes caused by a severe infection by promoting the production of short-chain fatty acids (SCFAs), particularly acetate. This process helped maintain the integrity of intestinal epithelial cells (IECs) and prevented the translocation of *E. coli* O157:H7 Shiga toxin.^129^ SCFAs play a significant role in innate and intestinal immunity, as they are histone deacetylase (HDAC) inhibitors with anti-inflammatory results.^127^ They enhance the differentiation of Tregs and support anti-inflammatory responses, by this means reducing the severity of colitis.^130^ SCFA acetate, butyrate, and propionate administration to germ-free mice improved the expression of anti-inflammatory IL-10-producing Foxp3-expressing Tregs through HDAC inhibition.^131^

 Moreover, butyrate increases IL-18 expression in epithelial cells, increases IL-10 expression in dendritic cells (DCs) and macrophages, allows them to boost the differentiation of Tregs, and protects against colitis.^132^ Thus, SCFAs influence both immune and non-immune cells in the intestine, playing a key role in regulating intestinal homeostasis. Acetic acid, propionic acid, and pentanoic acid significantly decreased the levels of norepinephrine, 5-hydroxyindole acetic acid (5-HIAA) and 5-HT in the hypothalami of depressed mice. SCFAs exemplify the signature hormones of the gut microbiota. Postbiotics can serve as connections between the gut microbiota and its various functions through traditional endocrine signaling. For instance, SCFAs have the ability to regulate the secretion of enteroendocrine 5-HT,^133^ which is a vital neurotransmitter at multiple levels of the microbiota–gut brain (MGB) axis.^134^ SCFAs, such as acetic acid, butyric acid, and propionic acid, can enter the circulatory system, and the gut microbiota may indicate to the host brain in this way.^135^

###  Mood management by GABA

 Gamma-aminobutyric acid (GABA) is an essential inhibitory neurotransmitter in the central nervous system, playing a vigorous role in decreasing neuronal excitability and adjusting muscle tone.^136,137^ Dysfunction of the central GABA system is related to anxiety spectrum disorders.^138,139^ In both animal and human research, substances that enhance GABA receptor activity typically exhibit anxiolytic effects, while those that inhibit these receptors tend to induce anxiety-like behaviors.

 Additionally, the gut microbiome can synthesize the neurotransmitter gamma-aminobutyric acid (GABA), which can then interact with and influence the central nervous system (CNS) through the enteric nervous system (ENS). This allows the gut microbiome to potentially modulate the gut-brain axis, the reciprocal communication pathway linking the gastrointestinal tract and the brain.^140^ Moreover, GABA has antidepressant,^141^ antidiabetic,^142^ antihypertensive,^143^ neuroprotective,^144^ cardiovascular regulatory,^145^ and anticancer^146^ effects. The biosynthesis of GABA in probiotic lactic acid bacteria was performed through the enzyme glutamate decarboxylase with L-glutamate as a substrate.^147^ The GAD system is an amino acid-dependent mechanism that helps maintain intracellular pH balance by facilitating the biosynthesis of GABA, contributing to acid resistance.^148^ As the main inhibitory neurotransmitter in the mammalian central nervous system, GABA modifies the function of growth hormones, development of the plasma concentration, and protein synthesis in the brain.^149^ GABA (201.78 µg/mL) was produced in MRS broth medium by *L. plantarum* K154, which was isolated from Kimchi.^150^ Additionally, *L. brevis* F064A, isolated from Thai fermented sausage, presented a high GABA content of 2.85 ± 0.10 mg/mL.^151^ Furthermore, yogurt containing *L. paracasei* had the maximum gamma-aminobutyric acid (GABA) content (99.63 μg/mL).^152^

###  Mood management by dopamine

 Dopamine is a crucial neurotransmitter responsible for the brain’s reward system, remarkably influencing feelings of pleasure and motivation.^147,153^ This hormone is related to happiness and regulates mood. Once, one individual feels a physical attraction towards another, the activation of dopamine and serotonin and the production of oxytocin increase. Oxytocin production leads to reduced pain. These processes regulate emotion and disconnect the amygdala, the part of the brain that is active against negative emotions such as fear.^154^ The gut microbiome contains certain bacterial species, such as *Staphylococcus aureus*, *Escherichia coli*, *Bacillus cereus*, *Bacillus mycoides*, *Bacillus subtilis*, *Proteus vulgaris*, and *Serratia marcescens*, which have the ability to produce more than half of the body’s total dopamine, a key neurotransmitter. This production of dopamine by the gut microbiome may allow for potential modulation of dopaminergic signaling pathways, including those in the central nervous system.^155,156^ *Enterococcus* sp.can generate dopamine in a gastrointestinal-like environment in the presence of the dopamine precursor L-3,4-dihydroxyphenylalanine (L-DOPA). *E. faecium* may affect the host through dopaminergic pathways. The production of dopamine by *E*. *faecium* closely reflects the accessibility of L-DOPA. However, L-DOPA is commonly found in various raw plants and plant-based food products. Under alkaline conditions and high temperatures, L-DOPA can break down into different quinones.^157^

###  Mood Management by Serotonin

 Serotonin (5-hydroxytryptamine, 5-HT) interferes with a wide range of physiological functions by stimulating multiple receptors, and abnormalities in these receptor systems are connected with many psychiatric disorders, including anxiety, depression, cognition, psychosis, migraine, feeding, disorders of sexual functioning and sleep. In the human gut, serotonin is synthesized and secreted by entero-chromaffin (EC), which plays a role in adjusting contractile frequency during peristalsis and influencing metabolic processes.^158^ The gut microbiome can influence serotonin levels through multiple mechanisms. Postbiotics, the metabolic byproducts of probiotic bacteria, can increase the concentration of serotonin within the intestinal lumen. This is achieved by upregulating the expression of key serotonin synthesis enzymes, such as tryptophan hydroxylase-1 (TpH1), and by downregulating the serotonin transporter’s expression (SERT). Additionally, secondary metabolites produced by probiotic bacteria can interact with microbial and host receptors, potentially contributing to this effect.

 Furthermore, the gut microbiome can modulate serotonin levels in the central nervous system. Likewise, the gut microbiota can affect the expression of serotonin receptors, transporters, and the enzymes involved in serotonin synthesis and metabolism, such as tryptophan hydroxylase-2 (TpH2) and monoamine oxidase (MAO). These interactions allow the gut microbiome to exert a broader influence on serotonergic signaling pathways throughout the body.^159^ Compared with germ-free mice, mice with integrated human gut microbiota have higher colonic levels of serotonin. Additionally, the mRNA levels of Tph1, which encodes the key enzyme responsible for serotonin synthesis,^160^ and chromogranin A (CHGA), which adjusts an indicator for serotonin secretion, are increased. An EC model cell line revealed that the intensification in Tph1 mRNA was induced by acetate and butyrate.^161^ A previous study indicated that butyrate could release unbalanced absorptive colonic motor activity in germ-free mice via the intervention of amended serotonin levels.^162^ Similarly, postbiotic acetate derived from *B. dentium* was demonstrated to promote colonic levels of serotonin. Interestingly, scientists have also shown that mono-colonized mice exhibit amplified expression of SERTs and receptors in the gut, as well as augmented expression of the 5-hydroxy tryptamine receptor 2A (Htr2a) receptor in the hippocampus.^71^ Increased intestine SERT expression has also been realized using *L. reuteri* and the supernatants of *Enterococcus faecium, Enterococcus faecalis* and *Bacillus subtilis*.^163^ Furthermore, the *E*. *coli* strain Nissle 1917 elevates host serotonin levels in a dose-dependent manner, which seems to be independent of all secondary metabolites (postbiotics) previously associated with regulating serotonergic gene expression.^164^ Spore-forming bacteria are sufficient to stimulate serotonin synthesis in EC cells, and these outcomes are mediated by postbiotics. In particular, bile acid chelates, butyrate, deoxycholate, p-aminobenzonate, and tyramine can augment Tph1 mRNA and colonic serotonin. Importantly, direct use of postbiotics only prompted temporary boosting of colonic serotonin levels, whereas colonization with spore-forming bacterial cells led to long-term elevation.^165-167^ Spore formation by *T. sanguinis *increases intestinal serotonin.^168^

###  Mood management by tryptophan

 Tryptophan is known for having a key role in the composition of neurotransmitters such as melatonin, niacin, and serotonin.^169^ Tryptophan cannot be produced by the human body and must be acquired through diet or released during protein breakdown. This essential amino acid plays a key role in various metabolic processes and serves as an important factor influencing mood, cognition, and behavior.^170^ Tryptophan serves as a precursor for the production of various important molecules, such as proteins, the aminergic neurotransmitter serotonin (5-hydroxytryptamine, 5-HT), and nicotinamide adenine dinucleotide (NAD/NADH). The conversion of tryptophan into these compounds occurs through the kynurenine biosynthetic pathway. The initial step in this pathway is the oxidation of tryptophan, which is catalyzed by enzymes like l-tryptophan 2,3-dioxygenase, IDO-1, or IDO-2, leading to the synthesis of the first stable metabolite, kynurenine.^171^

 The relationship between probiotic supplementary and the alteration of serotonin levels in patients undergoing combination antiretroviral therapy (cART) has been investigated. Researchers found that after 6 months of probiotic supplementation, there was a concurrent shrinkage in the levels of fecal tryptophan and an escalation in serum serotonin levels. It should be pointed out that only a small fraction, less than 1%, of the available tryptophan is actually converted into serotonin (5-HT) within the brain.^172^ Furthermore, the influence of probiotic supplementation on depression significantly reduces the kynurenine/tryptophan ratio, leading to a decrease in the BDI score.^173^ In comparison to the placebo, supplementation with *Lactobacillus plantarum* DR7 enhanced the serotonin pathway, evidenced by reduced levels of plasma dopamine β-hydroxylase (DBH), tyrosine hydroxylase (TH), indoleamine 2,3-dioxygenase, and tryptophan 2,3-dioxygenase, alongside increased expression of tryptophan hydroxylase-2 and 5-hydroxytryptamine receptor-6. Meanwhile, the dopamine pathway remained balanced due to consistent expression of TH and DBH over the 12-week period.^174^

###  Mood management by oxytocin

 Oxytocin is a neuropeptide hormone primarily synthesized in the paraventricular and supraoptic nuclei of the hypothalamus in the brain. This hormone is then stored and subsequently released into the general bloodstream. Oxytocin is well-known for its involvement in a variety of psychological and behavioral processes, such as those associated with love, happiness, and trust. These psychoactive effects are linked to various social and emotional experiences, including romantic attachment, sexual activity, and parental behaviors, through nurturing, pair-bonding, and cooperation.^175^ Oxytocin functions together with the hypothalamic‒pituitary‒adrenal stress axis to repress glucocorticoid stress-associated hormones and similarly reduce feelings of anxiety.^176^ Significantly, enhancing the skin’s resilience through improved wound healing capabilities helps safeguard against environmental threats and other potentially harmful agents.^177^

 In relation to mental health, oxytocin has anxiolytic properties, helping to reduce stress and promote feelings of well-being.^178^ Poutahidis et al. (2013)^124^ demonstrated that consuming *L. reuteri* in mouse models led to increased oxytocin levels in the host animals through a vagus nerve-mediated mechanism. This finding supports the existence of a gut-brain axis and underscores the role of gut bacteria in overall health. Even sterile lysates of *L. reuteri* were sufficient to boost oxytocin expression in the rodent brain, extending this effect to the hypothalamic-pituitary-adrenal (HPA) axis and ultimately reducing systemic levels of stress hormones.^179^ Similarly, pregnant and nursing female mice that consumed *L. reuteri* exhibited enhanced nurturing behaviors and produced more offspring compared to those on a fast-food diet or a control diet, further highlighting the profound impact of gut bacteria on physiological and behavioral outcomes.^180^ By activating the brain’s reward centers, oxytocin skews the nervous system towards pleasure and fosters a more positive outlook.^181^ Supplementation with *L. reuteri* enhances oxytocin levels in the hypothalamus, activates the mesolimbic dopamine reward pathway, and encourages prosocial behaviors.^182^ The consummation of purified *L. reuteri* in drinking water significantly increases the level of the neuropeptide hormone oxytocin in rats.^183^ A recent study investigated the synergistic effects of probiotics and oxytocin in individuals with Autism Spectrum Disorder (ASD) by consumption of *Lactobacillus plantarum* PS128 probiotic or placebo fosr 16 weeks. Outcomes showed improvements in the Social Responsiveness Scale (SRS) Aberrant Behavior Checklist (ABC), and the Clinical Global Impression (CGI) Improvement compared to the placebo.^184^ These findings reveal the astonishing role of oxytocin as a postbiotic involving mental, social, and physical health, with extensive possible benefits for high-quality and healthy individuals.

## Posbiotic–Brain Signaling

**Figure 2:**
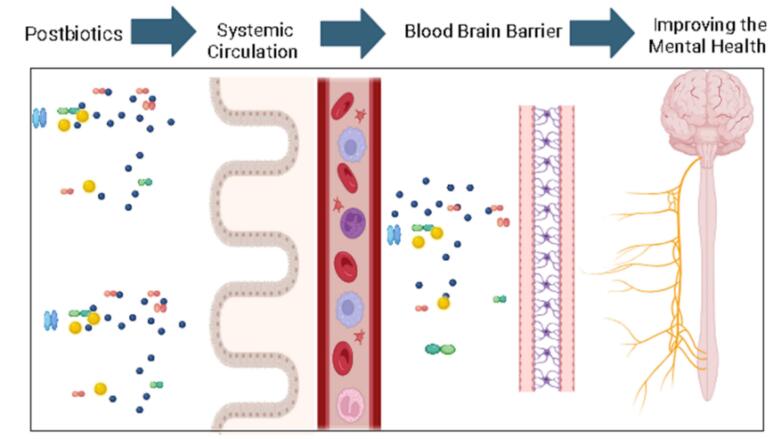
Interactions among probiotics, the blood‒brain barrier, and mental health

 The immune, metabolic, neuroendocrine, autonomic and enteric nervous systems (ENS) are known channels of gut–brain communication (Figure 2). Gut microbes produce postbiotics such as acetylcholine, noradrenaline, dopamine, and GABA I.^185^ The walls of the gut consist of the enteric nervous system and are primarily responsible for the motility of neurotransmitters and postbiotics, including short-chain fatty acids.^186^

###  Bacteria–Enteric Nervous System Signaling

 Neurodevelopment is a multifaceted process shaped by a combination of internal and external influences. The autonomic nervous system (ANS) and the enteric nervous system (ENS) play crucial roles in the neural regulation of gastrointestinal function. The physiological conditions of the gut, including acidity, nutrient levels, osmolality, and pain, are transmitted by the ANS to the brain.^187^ The enteric nervous system (ENS) is an awful complex network of neurons and supporting cells that is commonly known as the “second brain” due to its capacity of independently control the digestive system. The ENS is responsible for regulating many different functions in the gut, including the contraction and relaxation of smooth muscle, the secretion of digestive enzymes and fluids, and the control of blood flow and immune function. Bacteria are also known to interact with the ENS in several ways.^188^ Some species of bacteria are capable of producing neurotransmitters postbiotics that can activate or inhibit neurons in the ENS.^189^ These neurotransmitters, including dopamine, serotonin, and gamma-aminobutyric acid (GABA), are present in the central nervous system and play key roles in regulating mood, behavior, and various physiological processes.^190^

 The key factors influencing gut homeostasis are enteric neurons and enteric glial cells (EGCs), the second of which are discovered within the smooth muscle layer and the lamina propria of the mucosa.^191,192^ Therefore, Changes in the gastrointestinal (GI) physiology and environment are often related to nutrient contents or the establishment of a microbial population within the gut lumen. The maturation of the mucosal immune system likely influences the postnatal development of the enteric nervous system (ENS), indicating that both the gut environment and immune system are crucial during this phase of ENS growth.^187^ While the GI tract usually connects with CNS via the parasympathetic and sympathetic nervous systems (via the prevertebral ganglia, which are midline structures situated anterior to the aorta and vertebral column),^193^ the ENS functions as an intrinsic nervous system adept to individually controlling maximum physiological functions of the GI tract, including motility, reflexes, secretion, microcirculation, immune function, and the inflammatory process. These pieces of evidence have led to the conceptualization of a ‘brain in the gut’. However, both the ENS and the CNS communicate and affect each other. Specifically, vagal afferents transmit a variety of gastrointestinal signals to the central nervous system that reveal nutrient content, food intake and energy stores.

###  Vagal signaling

 Vagal signaling refers to the communication between the vagus nerve and various organs and systems in the body, including the gastrointestinal system, heart, lungs, and immune system. The vagus nerve sends signals from the brain to various organs, and it also receives signals from these organs, providing feedback to the brain.^194-196^ In the gastrointestinal system, vagal signaling is involved in regulating gastric acid secretion, intestinal motility, and nutrient absorption. The vagus nerve also plays a role in the regulation of appetite and satiety by sending signals to the brain about the presence of food in the stomach, and it is also responsible for the regulation of cardiovascular function.^197^ Vagal primary afferents are responsible for innervating both the muscular and mucosal layers of the gastrointestinal tract. The celiac branch specifically supplies the intestines, extending from the proximal duodenum to the distal section of the descending colon. The vagus nerve has its highest concentration of innervation in the proximal regions, yet it still plays a significant role in the colon.^198^ Gut hormones and regulatory peptides, including cholecystokinin (CCK), ghrelin, glucagon-like peptide-1 (GLP-1), and peptide tyrosine tyrosine tyrosine (PYY),^199,200^ which affect energy balance and the regulation of food intake, target chemosensitive receptors.^201^ Vagus can cause a reduction in anxiety- and depression-associated behaviors.^202^ *Lactobacillus gasseri* NK109 consumption suppresses IL-1β expression in elicited macrophages, leading to decreased cognitive impairment and depression in mice caused by celiac vagotomy.^203^ Moreover, Tanida et al. verified that intraduodenal injection of *L. johnsonii* La1 decreased renal sympathetic nerve activity and blood pressure while increasing gastric vagal nerve activity.^204^ Correspondingly, the consumption of a probiotic yogurt for four weeks increased the activity of the vagus nerve and improved mood in healthy volunteers.^205^

###  Short-Chain Fatty Acid Signaling

 Macronutrients such as plant polysaccharides (prebiotics) cannot be digested in the human gut. Although these substances are commonly found in our diet, humans lack the enzymes required for their digestion, which are instead provided by probiotics.^206^ The metabolism of these fibers results in the production of postbiotics such as short-chain fatty acids (SCFAs), including acetate, butyrate, propionate, and lactate.^207^ On the basis of these findings, Injections of sodium butyrate at a dosage of 200 mg/kg body weight in rats have been shown to elicit antidepressant effects, enhancing serotonin neurotransmission in the central nervous system and promoting the expression of brain-derived neurotrophic factor (BDNF).^208^ The role of butyrate as an epigenetic modifier is more probable than its function as an agonist at free fatty acid receptors (FFARs), particularly since there are relatively few FFARs present in the brain.^209^

 Nevertheless, SCFAs exhibit pleiotropy-independent effects generated by a single gene, and they either directly affect the mucosal immune system or promote the hypothalamic‒pituitary‒adrenal (HPA) axis.^210^ This discovery indicates that gut microbiota significantly influences the conversion of tryptophan into serotonin within enterochromaffin cells, which are specialized cells in the gut responsible for secreting serotonin. Specific metabolites, including indole and its derivatives, may affect how much tryptophan is available for serotonin production. Consequently, alterations in the gut microbiota’s composition could alter serotonin levels, potentially impacting mood.^211^ A study linked the role of native spore-forming bacteria in the gut to the regulation of serotonin production in enterochromaffin cells by bacteria, even though the precise mechanism governing this process was not understood at the time.^212^ Significant changes were observed in various tryptophan metabolites, particularly indole-containing compounds like indole-3-propionic acid (IPA) and indoxylsulphate. These metabolites were absent in germ-free mice, indicating that their production relies entirely on gut bacteria. Galactooligosaccharide (GOS), a key component found in breast milk, can be fermented by Bifidobacterium species to generate short-chain fatty acids (SCFAs).^213^ Thus, postbiotics, including SCFAs, have various amending functions, and their special effects on host physiology and immunity carry on to be investigated.^214^ Short-chain fatty acids (SCFAs) are essential for supporting gut health, regulating metabolism, and managing appetite. They can affect feelings of fullness by impacting the production of leptin, a hormone secreted by white adipose tissue. Disruptions in leptin signaling are linked to issues such as excessive eating, obesity, infertility, and immune dysfunction.

 SCFAs play a significant role in various metabolic and immune functions, with their concentrations in the gut varying between 20 to 140 mM. This variability is influenced by factors such as the host’s microbial makeup, dietary fiber intake, intestinal transit time, and metabolic processes.^215,216^ Short-chain fatty acids (SCFAs) generated by the gut microbiota can be detected and responded to by various receptors present in the host. These include the peroxisome proliferator-activated receptor gamma (PPAR-γ) and a class of G protein-coupled receptors (GPCRs), such as GPR41, GPR43, and GPR109. The binding of SCFAs to these receptors can modulate intracellular signaling pathways, such as those involving cyclic adenosine monophosphate (cAMP) and calcium levels. This receptor-mediated activity also leads to the stimulation of extracellular signal-regulated kinases (ERK1/2), which are imperative components of cellular signaling cascades. Furthermore, the presence of SCFAs has been observed to induce hematopoietic alterations, resulting in improved production of myeloid cells. This, in turn, can promote the clearance of systemic infections and potentially influence allergic processes within the host. This multifaceted mechanism of action highlights the complex ways in which the gut microbiome, through the production of SCFAs, can modulate various physiological and immune-related processes within the host organism.^217,218^

###  Immune system signaling

 Gut bacteria have a marked effect on balancing gut permeability, intestinal motility, and mucosal immune function and controlling the enteric nervous system.^219^ Dysbosis, a disruption in the gut microbiota, can activate inflammatory pathways, leading to an impaired immune system and disorders including inflammatory bowel disease and rheumatoid arthritis.^220,221^ Emerging research suggests that the gut microbiota, the various community of microorganisms be located in the intestines, can influence not only the immune cells found within the gut, but also those present in the brain. This reciprocal interaction between the gut and the brain holds significant consequences for our understanding of the body’s response to various neurological conditions. It has been observed that the stimulation of the immune system, both in the gut and in the brain, is associated with changes in the brain’s feedback to injury, the advancement of neuroinflammation, and alterations in neurogenesis (the creation of new brain cells) and neural plasticity (The brain’s capacity to adjust and evolve over time). This interplay between the gut microbiome and the brain’s immune system suggests that the microbiota may play a crucial role in modulating the brain’s reaction to various insults and disease processes.^222^

 One way in which postbiotics influence the immune system is by modulating the neuro-signal pathway, which is involved in the management of immune responses and inflammation.^223^ Furthermore, postbiotics stimulate the growth of beneficial gut bacteria population, which can further develop immune function.^224,225^ Postbiotics stimulate the production of anti-inflammatory compounds and help regulate the immune response, which can diminish the risk of infection and inflammation. Heat-inactivated *B. longum* reduces the acute inflammatory response by triggering pathways related to innate immune function.^226^ Similarly, heat inactivation in the cell supernatant of *Lactobacillus delbrueckii* CIDCA reduced the number of neutrophil cells infiltrating the small intestinal mucosa and recovered the intestinal epithelium architecture impaired by 5-fluoro uracil (5-FU).^227^ Activation of the immune system in both the gut and the brain is closely tied to the body’s response to neuroinflammation, brain injury, and deviations in neurogenesis and plasticity.^222^ Weakening in this two-way communication is involved in the etiopathogenesis of psychiatric and neurodevelopmental diseases and disorders, including autism disorders, as well as comorbidities related to gastrointestinal diseases, including inflammatory bowel diseases, where dysbiosis is commonly observed.^223^

## Discussion

 Emotional regulation is a critical psychological process that enables individuals to manage and respond appropriately to emotional experiences. This process involves initiating, inhibiting, or modifying emotional feelings and behaviors to support adaptive functioning. Deficits in emotional regulation are associated with various psychological disorders, including depression, anxiety, and borderline personality disorder.^228,229^ Effective emotional regulation promotes mental well-being by reducing the physiological effects of stress and improving interpersonal relationships. Strategies such as mindfulness, relaxation, and cognitive reappraisal are commonly used to enhance emotional regulation. This process is essential for maintaining mental health and fostering resilience.

 Neural pathways play a pivotal role in the process of emotional regulation, involving multiple brain regions. The angular gyrus, superior temporal gyrus, and supplementary motor area process information from the frontal cortex during emotional regulation.^230^

 The ventrolateral prefrontal cortex appraises emotional situations, relaying information to the dorsolateral prefrontal cortex, where regulation occurs. The anterior middle cingulate cortex integrates emotional information and contributes to generating emotional responses.^230^ These interconnected regions facilitate the modulation of emotional and behavioral reactions. Understanding these neural mechanisms provides insights into the brain’s role in emotional regulation.

 The gut-brain axis serves as a key mechanism through which postbiotics influence mental health. This bidirectional communication system connects the central nervous system with the gastrointestinal tract, involving the autonomic, enteric, neuroendocrine, and immune systems.^231,232^ Postbiotics, metabolic byproducts of probiotics, modulate this axis to promote emotional and cognitive health. These compounds integrate physiological, emotional, and cognitive processes through complex signaling pathways. The gut-brain axis is crucial for understanding the therapeutic potential of postbiotics in mental health disorders.

**Figure 3:**
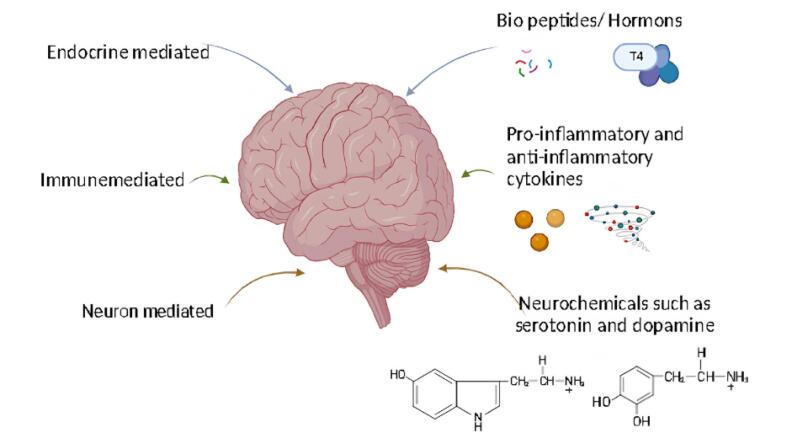
Postbiotics’ roles in brain signaling include bioactive peptides, hormones, dopamine, GABA, epinephrine, norepinephrine, cytokines, antiadhesion, gut barrier improvements, 5-HT, and neurochemicals

 Postbiotics, such as gamma-aminobutyric acid (GABA) and short-chain fatty acids (SCFAs), exert significant psychobiotic effects on mental health. GABA, an inhibitory neurotransmitter produced by lactic acid bacteria, regulates mood, sleep, and memory through interactions with GABAA, GABAB, and GABAC receptors.^186,233^ SCFAs, including acetate, butyrate, and propionate, modify cellular metabolism, support epithelial barrier integrity, and modulate neurotransmission. Other microbial metabolites, such as dopamine and oxytocin, influence reward behavior and mood regulation.^234^ These compounds collectively enhance mental health outcomes (Figure 3). Postbiotics represent a promising avenue for novel therapeutic interventions.

 The gut microbiome modulates the hypothalamic-pituitary-adrenal (HPA) axis, a critical component of the stress response. Dysbiosis in the gut microbiome can lead to sustained HPA axis activation, contributing to stress-related disorders. Postbiotics, by restoring microbial balance, may help re-establish HPA axis homeostasis. This modulation reduces cortisol levels and mitigates stress responses, supporting emotional regulation. Disruptions in the gut microbiome are also linked to increased pro-inflammatory cytokines, which are associated with psychiatric disorders.^235-239^ Targeting the HPA axis through postbiotics offers potential for managing mood disorders.

 Understanding these interactions is vital for developing targeted interventions. This integrative approach holds promise for improving mental health outcomes and fostering emotional resilience. Postbiotics are characterized by their unique properties, making them suitable for therapeutic applications. Comprising metabolites, cell wall fragments, and fermentation products, postbiotics are stable at room temperature, unlike live probiotics.^240,241^

 This stability enhances their commercial viability and ease of incorporation into treatment regimens. Their versatility makes them applicable to diverse populations, including those with mental disorders. The stability of postbiotics positions them as valuable candidates for innovative therapies.

 The safety profile of postbiotics supports their use in clinical settings. Toxicological studies show low cytotoxicity and genotoxicity in human cell lines, with minimal adverse effects.^240-242^ These compounds are well-tolerated, even in vulnerable populations like cancer patients. Mild effects, if any, are typically transient, indicating a favorable safety profile. Ongoing research continues to validate the safety of postbiotics for regular consumption. This safety profile enhances their potential as therapeutic agents.

 Regulatory perspectives on postbiotics vary, influencing their global application. The FDA and EFSA do not classify postbiotics as food additives or novel foods, simplifying pre-market evaluations.^243,244^ However, adherence to Good Manufacturing Practices (GMP) is critical to prevent microbial contamination during production. Compliance with local regulations ensures product safety and efficacy. These regulatory considerations are essential for the safe integration of postbiotics into health products.

 Individual variability in response to postbiotics is a crucial factor for their clinical use. Factors such as age, health conditions, and medications can influence responses to postbiotics.^243,244^ While generally safe, individuals with specific health concerns should consult healthcare professionals before use. This personalized approach optimizes therapeutic outcomes. Tailoring postbiotic interventions to individual needs enhances their effectiveness in mental health treatment.

 The long-term effects of postbiotics, particularly in mental health and oncology, warrant further investigation. Current evidence supports their safety for regular consumption, but prolonged use in specific populations, such as those with mental disorders, remains underexplored.^240,241^ Continued research is needed to understand the implications of extended use. Exploring these effects could unlock new therapeutic applications, including in oncology. Further studies will strengthen the evidence base for postbiotics’ clinical benefits.

 The interplay between gut microbiota, postbiotics, and neural circuits highlights the complexity of mental health regulation. Microbial metabolites, including serotonin, dopamine, and norepinephrine, modulate neurotransmission, synaptic plasticity, and cognitive function.^234^ These compounds influence brain health through diverse biochemical pathways, including enteroendocrine hormones and cytokines. Ongoing research continues to elucidate these mechanisms, emphasizing the gut microbiome’s role in mental health. This integrative approach offers promising avenues for improving emotional resilience and mental health outcomes.

## Conclusion

 In summary, this review highlights the emerging significance of postbiotics as pivotal modulators of mental health, particularly in the context of depression, psychosis, and mania. The accumulating evidence suggests that these microbial metabolites exert a profound influence on mood regulation, anxiety modulation, and cognitive function through their interactions with key neurotransmitter systems and their capacity to modulate neuroinflammatory pathways. Given the increasing recognition of the gut-brain axis, it is essential to undertake further empirical investigations to substantiate these findings and elucidate the underlying mechanisms through which postbiotics mediate their psychological effects.

 Future research should prioritize well-designed clinical trials assessing the efficacy of postbiotic interventions across diverse populations and a spectrum of mental health disorders. Incorporating postbiotics into existing therapeutic frameworks may provide innovative strategies to enhance patient outcomes and improve the quality of life for individuals affected by mental health conditions. This review advocates for a multidisciplinary approach that integrates microbiology, psychiatry, and nutritional science, thereby maximizing the therapeutic potential of postbiotics in mental health care. By advancing our understanding of the complex interplay between gut microbiota and psychological well-being, we can pave the way for novel and effective treatment modalities within the field of mental health.

## Competing Interests

 The authors declare no competing interests.

## Data Availability of Statement

 The data supporting the findings of this study are available within the article and its tables. Additional data may be available from the corresponding author upon reasonable request.

## Ethical Approval

 This study is a literature review and did not involve human or animal research. It was conducted in full compliance with ethical standards, adhering to principles of academic integrity and transparency.
